# Determining the optimal distal resection margin in rectal cancer patients by imaging of large pathological sections: An experimental study

**DOI:** 10.1097/MD.0000000000038083

**Published:** 2024-05-24

**Authors:** Shuhan Lin, Jie Wei, Hao Lai, Yazhen Zhu, Han Gong, Chengjiang Wei, Binglin Wei, Yinxiang Luo, Yi Liu, Xianwei Mo, Hongqun Zuo, Yuan Lin

**Affiliations:** a Hepatological Surgery Department, Guangxi Guigang People Hospital, Guigang City, Guangxi Autonomous Region, China; b Colorectal Surgery, Guangxi Medical University Cancer Hospital, Nanning, Guangxi Zhuang Autonomous Region, China; c Guangxi Clinical Research Center for Colorectal Cancer, Nanning, Guangxi Zhuang Autonomous Region, China; d Experimental Research Department, Guangxi Cancer Hospital, Nanning, Guangxi Autonomous Region, China.

**Keywords:** distal resection margin, large pathological section, rectal cancer, sphincter-sparing surgery

## Abstract

**Objective:**

To determine the distal resection margin in sphincter-sparing surgery in patients with low rectal cancer based on imaging of large pathological sections.

**Methods:**

Patients who underwent sphincter-sparing surgery for ultralow rectal cancer at Guangxi Medical University Cancer Hospital within the period from January 2016 to March 2022 were tracked and observed. The clinical and pathological data of the patients were collected and analyzed. The EVOS fluorescence automatic cell imaging system was used for imaging large pathological sections. Follow-up patient data were acquired mainly by sending the patients letters and contacting them via phone calls, and during outpatient visits.

**Results:**

A total of 46 patients (25 males, 21 females) aged 27 to 86 years participated in the present study. Regarding clinical staging, there were 9, 10, 16, and 10 cases with stages I, II, III, and IV low rectal cancer, respectively. The surgical time was 273.82 ± 111.51 minutes, the blood loss was 123.78 ± 150.91 mL, the postoperative exhaust time was 3.67 ± 1.85 days, and the postoperative discharge time was 10.36 ± 5.41 days. There were 8 patients with complications, including 3 cases of pulmonary infection, 2 cases of intestinal obstruction, one case of pleural effusion, and one case of stoma necrosis. The longest and shortest distal resection margins (distances between the cutting edges and the tumor edges) were 3 cm and 1 cm, respectively. The minimum length of the extension areas of the tumor lesions in the 46 images of large pathological sections was 0.1 mm, and the maximum length was 15 mm. Among the tumor lesions, 91.30% (42/46) had an extension area length of ≤5 mm, and 97.83% (45/46) had an extension area length of ≤10 mm. The length of the extension zone was not related to clinical pathological parameters (*P* > .05).

**Conclusion:**

In the vast majority of cases, the distal resection margin was at least 1 cm; thus, “No Evidence of Disease” could have been achieved. Additional high-powered randomized trials are needed to confirm the results of the present study.

## 1. Introduction

Rectal cancer (RC), a disease in which a malignant tumor is formed in the digestive system, is currently among the top 5 cancers in terms of morbidity and mortality rates.^[1,2]^ In nearly 70% of rectal cancer cases, the tumor occurs in the low part of the anal verge,^[3,4]^ and maximizing the protection of the patient’s physiological functions while treating the disease is a challenge for surgeons.^[5,6]^ The implementation of total mesenteric resection and preoperative radiotherapy and chemotherapy significantly reduces the local recurrence rate of the disease and improves the survival rate of rectal cancer patients.^[7]^ However, the prognosis was not significantly improved in patients with ultralow rectal cancer compared to patients with middle and upper rectal cancers.^[8]^

Abdominoperineal combined resection was once a surgical procedure for the treatment of ultralow rectal cancer, but it seriously affects the quality of life of patients.^[9]^ With the development of preoperative neoadjuvant radiotherapy and chemotherapy, the technology of laparoscopic surgery continues to improve, and with the evolution of surgical instruments,^[10]^ the success rate of sphincter-sparing surgery (SSS) for ultralow rectal cancer has increased.^[11]^ However, there are still some controversies regarding the distal resection margin (the distance between the cutting edge and the tumor edge).^[12]^

An insufficient distal resection margin is one of the reasons for local recurrence.^[13]^ The literature suggests that a positive circumferential resection margin is the main risk factor for the local recurrence of rectal cancer.^[14]^ However, the distal resection margin is still controversial, and the conclusions arrived at by studies regarding it are inconsistent. As the distal resection margin in the vast majority of rectal cancer patients is generally <1 cm, most scholars believe that the distal resection margin can be <1 cm.^[15]^ However, some research articles report that the 5 mm distal resection margin has also achieved “No Evidence of Disease (NED)” and does not affect the local recurrence rate or the 5-year survival rate.^[15–17]^

The existing pathological detection methods use fixed-point sampling detection, which is not dynamic and coherent. A pathological detection technique that uses large pathological sections and can observe continuous changes in tissue sections has been developed in recent years. In the present study, the clinical data of patients with low rectal cancer who underwent SSS based on imaging of large pathological sections were analyzed, the distribution of the tumor cells and the morphological changes in different regions of the primary lesion of rectal cancer based on images of large pathological sections were observed, the tumor heterogeneity stages in different regions were compared, and the lengths of the tumor tissue extension areas were measured to determine the relationship between the distal resection margin and clinical pathological parameters in SSS for ultralow rectal cancer.

## 2. Materials and methods

### 2.1. Case collection

This study was approved by the Ethics Committee of Guangxi Medical University Cancer Hospital. The clinical data (e.g., gender, age, body mass index, pathology, surgical time, blood loss, tumor size, T stage, and N stage) of rectal cancer patients who underwent SSS at the Guangxi Medical University Cancer Hospital within the period from January 2016 to March 2022 were collected through a hospital medical record retrieval system. The subject inclusion criteria were as follows: cT1-3 stage rectal cancer patients with complete clinical and pathological data in the aforementioned hospital; without any other tumors or dysfunction in the heart, lungs, or liver; and fully understands the conditions of study participation and voluntarily signs an informed consent form. Those who did not meet these inclusion criteria were excluded from the study.

### 2.2. Collection and processing of pathological specimens

We first collected complete specimens of the patients’ primary lesions and transformed them into large pathological wax blocks. We washed the specimens with phosphate-buffered saline solution or sterile physiological saline and then cut them to ensure the integrity of the tumor tissue and the main tumor area. Thereafter, we fixed the intestinal tube, including the tumor tissue, around a foam plastic plate using foam thumbnails to prevent contraction and movement of the specimens. (Tumor incision and fixation are beneficial for the full penetration of formalin into the tissue to avoid necrosis caused by overnight placement after insufficient tissue fixation.) We then completely immersed the fixed intestinal tube into a specimen bag containing 10% neutral formalin solution and sent it to the pathology department.

Imaging of large pathological sections was carried out along the longitudinal axis of the intestinal canal, usually consisting of 5 to 10 consecutive sections. The overall morphology should include the complete proximal tumor distal region, which should be treated with a slide to prevent detachment. Five to ten 3- to 5-µm wax blocks were sliced consecutively using a large pathological microtome. Subsequently, hematoxylin eosin staining was performed. The image was obtained through panoramic scanning using Tissue FAXS software.

### 2.3. Statistical analysis

We performed a statistical analysis using IBM SPSS Statistics 23.0. Measurement data are presented as mean ± standard deviation (X ± SD). An unpaired double-sided *t* test was used for the comparison of 2 groups. A chi-square test was used to compare the differences between the measurement data. A 1-way analysis of variance was used to compare the mean values of multiple groups. Finally, the least significant difference test was used for the comparison of the 2 groups. Statistical significance was based on *P* < .05.

### 2.4. Follow-up

Follow-up data were acquired mainly by sending letters to the patients and contacting them via phone calls, and during outpatient visits. The last follow-up was on September 30, 2022. The maximum follow-up time was 75 months. All the patients were followed up successfully.

## 3. Results

### 3.1. Analysis of the patients’ basic conditions

Based on the medical records of the 46 patients (25 males, 21 females) aged 27 to 86 years who participated in the present study, there were 9, 10, 16, and 11 patients with stages I, II, III, and IV low rectal cancer, respectively. In this patient cohort, the T stage distribution was as follows: 11 patients had T1 + T2 stage cancer, while 35 patients were classified as T3. Eight patients underwent chemoradiotherapy.

Characteristics of the patients and surgical outcomes are presented in Table 1. One patient underwent laparoscopic intershipincteric resection (LISR), during which the distal margin was excised using an ultrasound knife; 2 patients underwent Miles surgery; the remaining patients underwent laparoscopic Dixon surgery, where the distal rectal transection was performed using a linear staple.

**Table 1:** characteristic of patients and surgical outcomes.

Variate	N = 46
Gender	
Male	25
Female	21
Age	
≥60	25
<60	21
Differentiation	
Medium-low differentiated	32
Well differentiated	14
Lymphatic metastasis	
Positive	25
Negative	21
T stage	
T1 + T2	11
T3	35
TNM stage	
I	9
II	10
III	16
IV	11
Operation time	273.82 ± 111.51 min
Blood loss	123.78 ± 150.91 mL
Time to first flatus	3.67 ± 1.85 d
Hospital stay	10.36 ± 5.41 d
Complications	
Pulmonary infection	3 (6.52%)
Intestinal obstruction	2 (4.35%)
Pleural effusion	1 (2.17%)
Stoma necrosis	1 (2.17%)
Postoperative intestinal perforation	1 (2.17%)

The mean operation time was 273.82 ± 111.51 minutes, the mean blood loss was 123.78 ± 150.91 mL, the mean time to first flatus was 3.67 ± 1.85 days, and the mean hospital stay was 10.36 ± 5.41 days. Eight patients (17.39%) had complications, including 3 cases of pulmonary infection (6.52%), 2 cases of intestinal obstruction (4.35%), 1 case of pleural effusion (2.17%), and 1 case of stoma necrosis (2.17%), all of which were cured through conservative treatment. Postoperative intestinal perforation occurred in 1 case (2.17%) and was cured through reoperation.

As of the follow-up deadline, 15 out of 46 patients (32.61%) had died of tumor recurrence, progression, and metastasis, with an average survival time of 25.33 ± 19.06 months. Among these patients, 6 patients were found with liver recurrence, 7 patients were found with multiple metastases, and 1 patient was found with intraabdominal lymphatic metastasis. No local recurrence was found in the recurrent patients.

### 3.2. Cellular and histomorphological observations of large pathological sections of the primary lesion in rectal cancer

**Figure 1.**
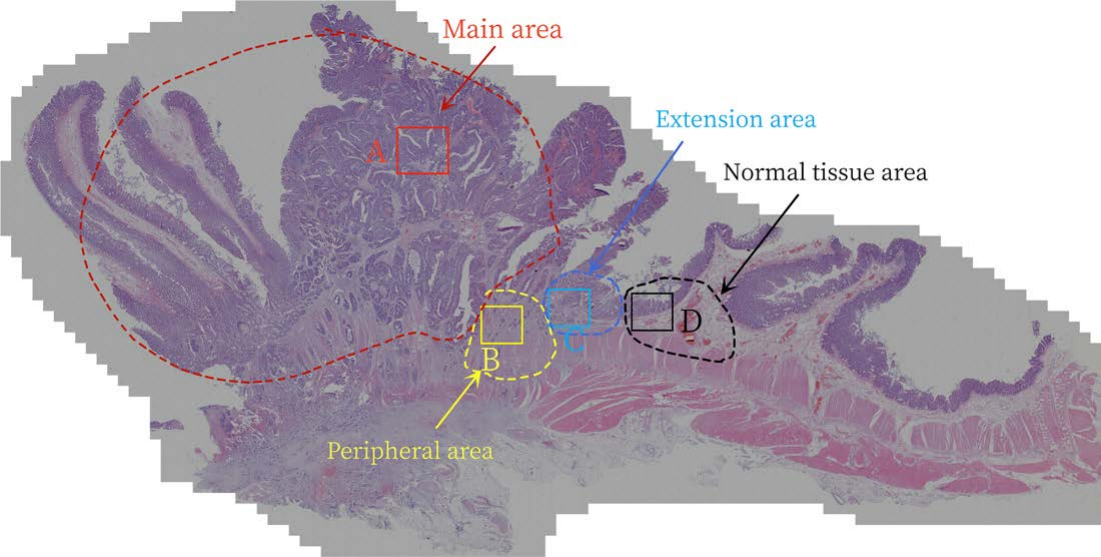
Distribution of the 4 zones in a large pathological section of rectal cancer: (A) main area; (B) peripheral area; (C) extension area; (D) normal tissue area.

**Figure 2.**
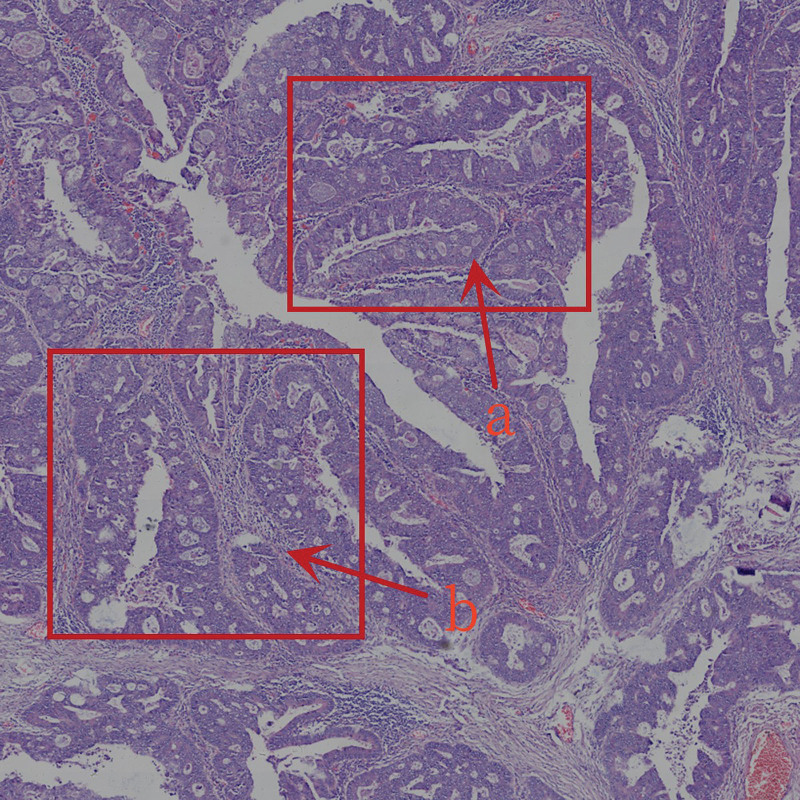
(A) Tumor cells and cancer nests in main area; (B) gland structure of the lesion is chaotic and damaged.

**Figure 3.**
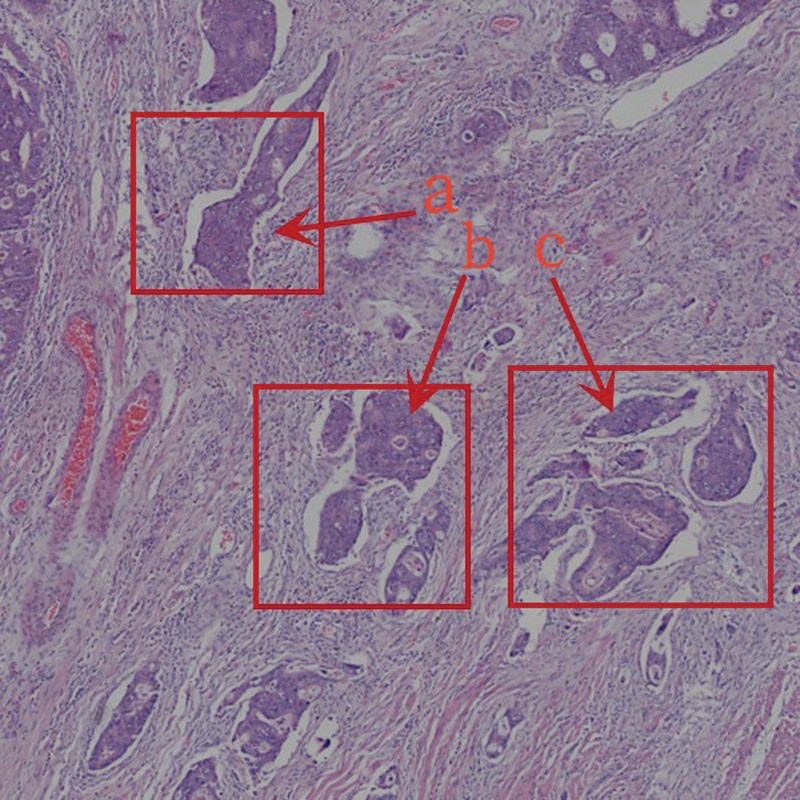
(A–C) Tumor cells in the peripheral area.

**Figure 4.**
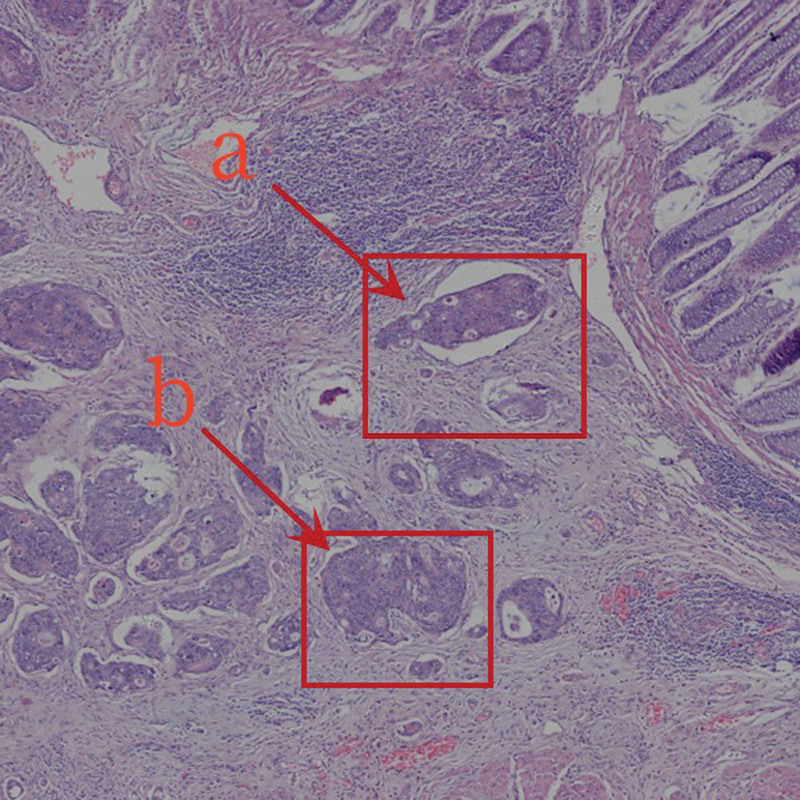
(A and B) Tumor cells in the peripheral area.

**Figure 5.**
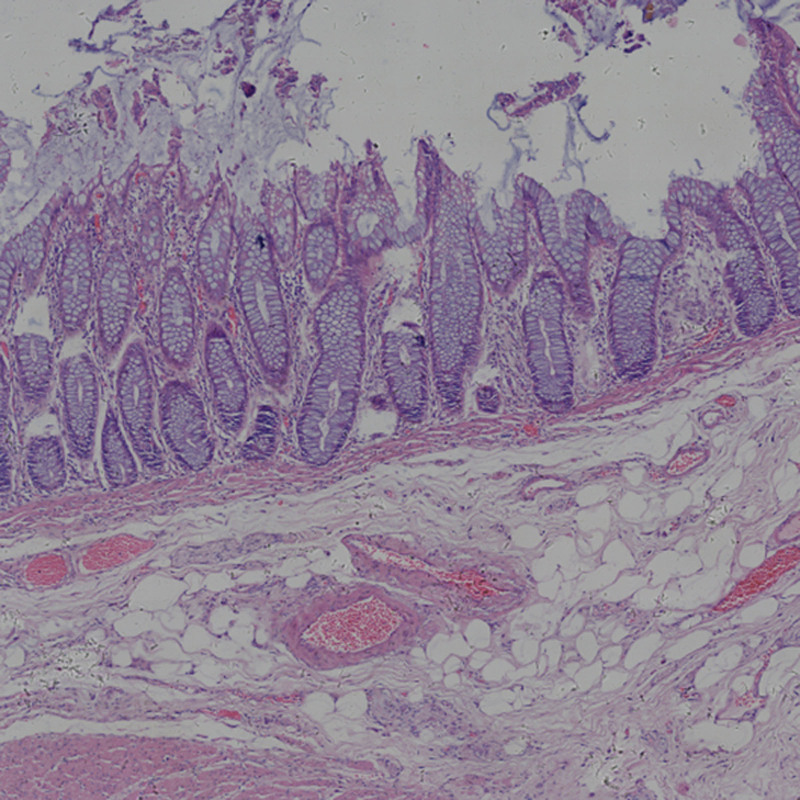
Peripheral area.

The Tissue Gnostics panoramic tissue scanning imaging system was used to obtain complete images of large pathological sections of the primary lesions. We divided each section into the following 4 zones based on the morphological characteristics (Fig. 1):

(1) *Main area (A).* The part with the most concentrated tumor tissue and structure under low magnification, where many tumor cells and cancer nests are densely distributed (Fig. 2A), squeezing and stacking each other, and the gland structure of the lesion is chaotic and damaged (Fig. 2B).

(2) *Peripheral area (B).* The area where the tumor cells (Fig. 3A–C) and structural continuity extend in the main lesion area under low-power microscopy.

(3) *Extension area (C).* At low magnification, the tumor tissue and cells (Fig. 4A and B) are distributed in scattered and discontinuous areas outside the continuous extension terminal of the tumor tissue and structure. The starting point is the boundary between the peripheral area and the extension area, and the endpoint is the farthest area of distribution of the tumor cells along the mucosal bottom layer.

(4) *Normal tissue area (D).* An area completely free of tumor cells and tissues (Fig. 5).

According to the 4 partitions/divisions, we observed the pathological tissue morphology of different regions. In the main area, we saw many tumor cells gathering, with the local structures disordered and irregular. The normal organization was disrupted, and the normal organizational structure was almost invisible. The glandular structure of the lesion was disordered and disrupted, with significant structural changes, and cellular heterogeneity was evident. The tumor cells in the extended area were scattered and irregularly present in normal tissue. No morphological changes such as rapid cell proliferation or accumulation could be seen in the field of vision.

### 3.3. Analysis of the extension zone lengths of the rectal cancer specimens

The longest and shortest distal resection margins measured 3 cm and 1 cm, respectively, with postoperative pathology indicating no cancer presence at the distal cutting edge. After surgery, all the primary lesions were observed through large pathological sections therein. The extension area at the distal end of the primary lesion was measured based on an image of a large pathological section. The shortest length of the extension area was 0.1 mm, and the longest was 15 mm. In the patient with a 15 mm extension area, patient, the distal resection margins was 2.5 cm, and no cancers residue was detected through pathology analysis. Postoperative pathology confirmed that all patients achieved R0 resection with negative distal margins.

Among the patients, 91.30% (42/46) had an extension area length of ≤5 mm, and 97.83% (45/46) had an extension area length of ≤10 mm. The results (Table 2) obtained in the present study suggest that the length of the extension zone in rectal cancer patients is not related to clinical pathological parameters such as gender, age, maximum depth of infiltration, TNM staging, degree of differentiation, tumor classification, tumor site, and tumor size (*P* > .05).

**Table 2:** Association between the extension zone length of the primary tumor and clinical parameters in 46 rectal cancer patients.

Parameter	N	Length of extension zone (mm)	*P*
Gender			
Male	25	1.92 ± 1.87	.07
Female	21	2.91 ± 3.70	
Age (y)			
≧60	25	2.2 ± 2.33	.14
<60	21	3.07 ± 2.78	
T stage			
T1 + T2	11	2.25 ± 1.78	.15
T3	35	2.70 ± 3.39	
Differentiated			
Medium-low differentiated	32	2.89 ± 3.47	.12
Well differentiated	14	1.91 ± 1.81	
TNM stage			
I	9	2.43 ± 1.87	.31
II	10	1.91 ± 3.58	
III	16	3.56 ± 3.82	
IV	11	1.49 ± 1.45	
Tumor site			
Below peritoneal reflexion	20	2.38 ± 2.74	.89
Above peritoneal reflexion	26	2.77 ± 3.34	
Tumor size			
<5 cm	30	2.81 ± 2.74	.90
≧5cm	16	2.20 ± 3.68	

## 4. Discussion

The R0 distal resection margin is the key to ensuring the radical resection of tumors.^[18]^ In the early 20th century, Miles proposed that the safe distance between the lower incisal margin and the edge of the tumor in SSS for rectal cancer should be at least 5 cm.^[19]^ With the development of total mesorectal resection, some scholars believe that the distal resection margin in rectal cancer should not be <1 to 2 cm to meet the requirements.^[20]^ Some research articles report that the local recurrence rate of rectal cancer is related to the distal resection margin.^[14,21]^ In a study that included 507 cases of rectal cancer, the 3 years local recurrence rates of the <1 cm, and >1 cm distal resection margins were 8% and 2%, respectively.^[22]^ Studies have also demonstrated that there is no statistically significant difference in the 5-year survival rate between distal resection margins of <0.5 cm, and 0.5 to 1 cm in SSS for rectal cancer patients.^[15,23]^ However, in low and even ultralow rectal cancer SSS, an increase in the distal resection margin means a decrease in the success rate of sphincter preservation,^[18]^ which will have a significant impact on the patient’s quality of life.

In the present study, 46 patients with rectal cancer were selected, and surgical specimens were collected from them for the imaging of large pathological sections. The EVOS FL Auto intelligent automatic fluorescence microscopy imaging system was used to perform full and multilayer scanning of large pathological sections and to obtain panoramic images of such sections. The distribution and structural characteristics of the cells in different regions of the large pathological sections were then analyzed, and the infiltration and cell distribution ranges of primary tumors in rectal cancer were clarified.

The results obtained from the examination of the large pathological sections indicate that the primary lesion of rectal cancer is divided into 4 different regions: the main area, the peripheral area, the extended area, and the normal tissue area. On the vast majority of pathological slides, a number of regular changes were seen. The tissue structure of the main area was disordered, and the mucosal layer and submucosal tissue were significantly damaged. The tumor cells gathered densely and infiltrated the deep mucosa and muscle layers in various forms. When the tumor cells extended outward from the point of origin, most of them first extended outward along the submucosal space. The distance of the extension along the submucosal layer was often farther than that toward the deep muscular layer.

The traditional view is that the longer the surgical margin, the greater the curative effect. According to the Chinese guidelines for the diagnosis and treatment of colorectal cancer, the distal resection margin in SSS for rectal cancer should be at least 2 cm.^[24]^ However, in some surgical procedures, increasing the distal resection margin can decrease the patient’s quality of life. The large pathological sections examined in the present study suggest a transitional area between the tumor and normal tissue, which we define as the extension area of the tumor. In this area, scattered and isolated cancer cells are generally visible. In clinical practice, whether there are residual cancer cells at the lower incisal margin is often used as a criterion for evaluating the effectiveness of radical tumor surgery. However, it is not clear whether the distal resection margin is related to the patient’s basic information, such as tumor size and T stage. Due to the lack of an intuitive quantitative basis for the distal resection margin, it is still a controversial matter, and a consensus still has to be reached regarding it. However, among the 46 cases of rectal cancer in the present study, the lengths of the tumor extension zones ranged from 1 to 15 mm, with 43 cases (93%) having lengths below 10 mm, and with only 2 cases having 10 mm lengths and only one case having a 15 mm length. This means that a 10 mm distal resection margin can basically ensure that the vast majority of patients will have no residual tumor at that margin. In our cohort, the distal margins were pathological negative. However, should the distal margin be deemed insufficient intraoperatively, immediate frozen section pathology is advised, followed by recommended additional resection.

Moreover, there was no significant correlation between the length of the extension zone and parameters such as patient gender, age, T stage, TNM stage, vascular tumor thrombus, and tumor size. The analysis results suggest that when deciding on the distal resection margin, more attention must be paid to protecting the patient’s postoperative physiological function than to adjusting the margin based on the patient’s clinical parameters. In SSS for low rectal cancer, it is necessary to preserve as much of the patient’s physiological functional structure as possible while ensuring the effectiveness of radical treatment.

According to the literature, surgical specimens are fixed with formalin contract by one-third compared to those before fixation, which can affect the measurement of the distal resection margin.^[16,25]^ Thus, before immersing a specimen in formalin, we used foam thumbnails to fix the intestinal tube, including the tumor tissue, around the foam plastic plate to prevent the contraction of the specimen and to ensure the accuracy of the measurement of the distal resection margin.

We believe that much clinical evidence has been found in the past few decades that the distal resection margin in radical tumor surgery is not as large as it can be. When it comes to surgical methods involving functional and structural destruction, it is necessary to preserve the patient’s physiological function as much as possible while ensuring a curative effect. In fact, the quality of life of tumor patients after treatment seriously affects their disease prognosis.

Previous studies have revealed that distal spread is really observed in stage II and III rectal cancer patients. One study, involving 610 rectal cancer specimens, found distal spread in only 1.2% of stage II and 5.1% of stage III RC patients, all within 1 cm.^[26]^ Multifactorial analysis also indicated that distal spread is not an independent risk factor for local recurrence.^[27]^ Instead, CRM involvement may have a more significant role in prognosis than distal spread.^[28]^ In the present study, based on our analysis of the patients’ clinical data, a distal resection margin >1 cm could have ensured a residual cancer-free effect in approximately 97.83% of the patients (45/46), which is consistent with many clinical reports on local recurrence rates in recent years. Therefore, in SSS for low or ultralow rectal cancer, it is necessary to shorten the patient’s distal resection margin as much as possible to avoid damaging important functional structures and to achieve the goal of preserving the anal sphincters, which are highly valued by clinical doctors. If necessary, intraoperative rapid freezing pathology can be conducted.

## 5. Conclusion

All the patients with low rectal cancer who participated in the present study underwent SSS under laparoscopy, with satisfactory results. In the vast majority of cases, the distal resection margin was at least 1 cm, and NED could have been achieved. Additional high-powered randomized trials are needed to confirm the results of the present study.

## Author contributions

**Data curation:** Shuhan Lin, Jie Wei, Han Gong, Chengjiang Wei, Xianwei Mo, Hongqun Zuo.

**Formal analysis:** Shuhan Lin, Jie Wei.

**Writing – original draft:** Hao Lai, Yazhen Zhu.

**Writing – review & editing:** Hao Lai, Yazhen Zhu, Hongqun Zuo, Yuan Lin.

**Validation:** Binglin Wei, Yinxiang Luo, Yi Liu.
